# Editorial

**Published:** 1864-12

**Authors:** 


### Editorial.

Chicago Medical Society.—The regular weekly meeting of this Society, on Friday evening last, was well attended; and the proceedings were interesting and profitable.

We select the following items as worthy of record:—*Triticum repens or Couch Gross.*—Dr. Waite called the attention of the Society to the efficacy of this article in relieving chronic irritation and inflammation of the bladder. He related two cases of long standing and great severity, which had been relieved by it. They were both in men past the middle period of life, and were well marked cases of chronic inflammation. The remedy was used in the form of infusion, made by putting a pint of boiling water on an ounce of the herb; of which a wineglassful may be taken every two, four, or six hours, according to the urgency of the symptoms.

*Chronic Diarrhœa of Camps.*—Dr. Peterson alluded to this disease for the purpose of stating that he had treated several cases with satisfactory success, by giving a combination of sub. nit. bismuth, sub. carb. ferri, &c., according to the following form:—

℞. Sub. Nit. Bismuth, ---------------------- ℈ij.

Sub. Carbonate of Iron, ---------------- ℈j.

Sulph. Morphine, ---------------------- 1gr.

Mix, and divide into eight powders; one of which may be taken every three, four, or six hours, according to the frequency of the evacuations.

*Hemorrhoids.*—Dr. H. Wanzer related a case of hemorrhoidal tumors of long continuance, and which was accompanied by such a degree of pain and spasmodic action in the sphincter ani as to be hardly endurable. Finding the tumors too large and indurated to remain reduced when carefully returned above the sphincter, he extirpated them by ligatures. The recovery was complete, and without any bad symptoms.

*Cases of Neuralgic Rheumatism.*—Dr. N. S. Davis read a report of several cases, which elicited some discussion; during which, the fact that cases of irregular and unusual attacks of a rheumatic nature, was made apparent. The cases, as reported, will be found in another part of this number of the EXAMINER. At a previous meeting, a question had arisen, whether a regularly educated physician, who had formerly violated the Code of Ethics, by newspaper and handbill advertising, in reference to the treatment of particular diseases, &c., but who, for four or five years past, had abandoned such practices and conformed his conduct to the ordinary rules of the profession, should be elected a member of the Society. The question was referred to a committee of three, who made the following report:—

The Committee appointed to report in reference to the admission of applicants for membership, who had at some previous time been guilty of violating that rule of Ethics relating to advertising, &c., would ask leave to report as follows:—

The section, in the established Code of Medical Ethics, relating to this subject, is as follows:—

"It is derogatory to the dignity of the profession, to resort to public advertisement, or printed cards, or handbills, inviting the attention of individuals affected with particular diseases—publicly offering advice and medicine to the poor *gratis*, or promising radical cures; or to publish cases and operations in the daily prints, or suffer such publications to be made; to invite laymen to be present at operations; to boast of cures and remedies; to adduce certificates of skill and success; or to perform any other similar acts. These are the ordinary practices of empirics; and are highly reprehensible in a regular physician."

The question referred to your Committee may be stated briefly in these words:—Is it consistent with the interests and honor of the Society to elect to membership any individual who had deliberately violated the first part of the foregoing rule, by advertising to cure particular diseases, both in the public prints and in handbills, &c.; but who, for one or more years previous to asking admission into the Society, had entirely discontinued such advertising, and manifested a sincere desire to conform his conduct to the established rules of the profession?

That the Society has a right to admit such an individual to membership, there can be no doubt.

If the Society was an incorporated one, intended to embrace all regular physicians, and to the members of which were secured certain legal rights and privileges, as is the case with the State and County Medical Societies in the State of New York, it might be the *duty* of the members to admit such an applicant, as is supposed in the question referred to the Committee. It was, at least, so decided *judicially* in a recent case, of a somewhat similar charcter, in connection with the Erie County Medical Society, in that State. But if the organization of the Society is, like ours, purely voluntary, and for the mutual improvement of its members, without legal existence or legal privileges, then certainly no member of the profession can claim admission as a right; and much less can it be claimed as a *duty* of the Society to elect such an applicant as is contemplated in the question referred to the undersigned for consideration. Under such circumstances, the question of admission or rejection becomes one of mere expediency, to be decided, not by the wishes of the one who may be proposed for membership, but by the influence it may have on the interests and honor of the Society itself. A physician, who has deliberately violated the established rules of Medical Ethics, and subsequently repents, and abandons such violations, should, like all other repentant sinners, be forgiven by his medical brethren, just as far as his repentance is known to them. But when a regularly educated physician, and graduate of a respectable medical school, causes to be published extensively in the newspapers in different localities, advertisements claiming superior success in the treatment of particular diseases, and sends handbills broadcast upon the community making the same pretensions, his name becomes indissoluably associated, in the professional mind, with *quackery*. And though he may subsequently withdraw his advertisements, and cease to distribute his circulars, and so far regulate his conduct by the rules governing all respectable physicians, as to satisfy his immediate neighbors in the profession, yet over all the territory reached by his previous advertisements, his name remains with its old association uncorrected. Hence any mere voluntary medical society or association, that should place such a name on its list of membership, would necessarily incur the odium, everywhere beyond the personal acquaintance of its members, of an association with charlatans and newspaper puffery. Your Committee, therefore, unanimously recommend that no such individuals be admitted as members of this Society, unless, in addition to the abandonment of their irregular practices, they make the fact of such abandonment as fully and extensively *public* as their previous advertisement and circulars have been.

All of which is respectfully submitted.

N. S. DAVIS,
A. H. THOMPSON,
JOHN REID.

---

MORTALITY OF CHICAGO.—The whole number of deaths in this City for each of the last five years, ending December 31st, 1864, is as follows:—

| 1860. | 1861. | 1862. | 1863. | 1864. |
|---|---|---|---|---|
| 2,056 | 2,069 | 2,575 | 3,522 | 4,060 |

The population of the City, during the same time, is officially reported as follows:—

| | |
|---|---|
| 1860, | 110,973 |
| 1862, | 138,835 |
| 1864, | 170,000 |

This indicates a ratio of one death in every *fifty-three* of the population, for the first three of the five years, and one in every *forty-one* for the remaining two years. If we compare these statistics with those on the same subject furnished by other large cities, either in this country or Europe, we shall find few, if any, of equal population, to present as low an average ratio of mortality for the last five years.

This very gratifying result is not owing to any superior hygienic regulations devised and enforced by the municipal authorities of Chicago, or to the absence of such causes as tend to deteriorate the health of cities generally.

But it is owing mainly to these circumstances, namely: first, our streets are nearly all broad, straight, and intersect each other at right angles; second, the nature of the soil is such that no families live in cellars or basements below the natural level of the earth's surface; and third, the broad lake on the east, and the open, unobstructed prairie on the west, causes the winds from either direction to sweep freely through every street and alley in the City. This unusual freedom of ventilation is, undoubtedly, the most important conservator of public health that our City enjoys. With the same causes existing, calculated to impregnate both the air and the water with impurities, if any considerable part of our population had been crowded into basements, or narrow, crooked streets, admitting of only imperfect ventilation, the ratio of mortality would have been greatly increased.

But, with all the advantages of broad streets and the greatest freedom of ventilation, the reader will not fail to notice a marked increase in the ratio of mortality during the last two years, as compared with the three immediately preceding. This, when the attendant circumstances are understood, is a fact of great interest to the students of *etiology* generally, as well as to the people of Chicago. It was not until the last two years that the blood and offal of the packing houses and their appendages mingled with the sewage matter of the Chicago river so freely as, not only, to furnish gaseous emanations sufficient to impregnate the whole atmosphere of the city, but also to taint the water along the lake shore to such an extent as to be returned through the hydrants into every house, nauseous both in taste and smell. The first special prevalence of disease following this state of things, which attracted attention, was a pretty severe epidemic of erysipelas, commencing in September, 1863, and continuing until the middle of the following winter. And sporadic cases of the same disease have continued to occur more frequently than formerly up to the present time. The dysenteries occurring during the autumn of both 1863 and 1864, were more obstinate and protracted than usual. And cases, both of diarrhœa and dysentery, were met with much more frequently than in former years, during all the winter of 1863-4, and have been, also, thus far, during the present winter. From the decline in the epidemic of erysipelas, just alluded to, up to the present time, cases of continued fever have been more numerous in proportion to the population than in previous years. They have also, especially during the last six months, presented much more prominently the characteristics of *Typhus*. Without alluding to other, and less important, changes in the character and prevalence of diseases, we cannot hesitate to attribute those already mentioned, to the direct influence of the decomposing animal matter that has so strongly impregnated both the air and water of our city for the last two years. This conclusion is also corroborated by the change in the ratio of mortality in the different seasons of the year. The mortality for each quarter is as follows:—

| | 1860. | 1861. | 1862. | 1863. | 1864. |
|---|---|---|---|---|---|
| 1st quarter, | 423 | 500 | 570 | 908 | 1060 |
| 2d do | 388 | 391 | 509 | 776 | 869 |
| 3d do | 613 | 728 | 849 | 996 | 1319 |
| 4th do | 477 | 470 | 645 | 842 | 806 |

A glance at this table will show that, during the last two years, the ratio of deaths has been much more equally distributed through the different quarters of the year, thereby indicating the existence of some more constantly acting cause, aside from the ordinary meteorological influences. But we have not time to pursue the subject further at present.

---

Profits of Medical Practice in Chicago.—Some of the daily newspapers in this city, having published a complete list of the taxable incomes of our citizens, of all classes, as returned to the office of the Assessor of Internal Revenue, for 1863, we had the curiosity to examine so much of the list as related to the incomes of members of our profession.

By an examination of the City Directory, we were able to identify the names of 120 regular physicians and surgeons, actually engaged in the practice of their profession in this city. Also 25 homœopaths, and about 20 advertising quacks. Of the first 120, we were able to identify only 41 as having returned any amount of taxable income, above the legal deductions, for the year 1863.

The amount of taxable income returned by the 41, whose names were on the printed list, ranged from $75 to $6041. Of the whole number 1 only reported over $5000; 4 between $3000 and $5000; 11 between $1000 and $3000; 16 between $500 and $1000; 9 below $500.

Of the 25 homœopathic practitioners, we identified the names of only 10, in the published list, as returning some amount of taxable income. Of these, 4 ranged between $1000 and $2000; 3 between $500 and $1000; and 3 less than $500. The highest in this list was $1888, and the lowest $214. Of the 20 or more puffing newspaper advertising quacks, under the title of *Doctor*, only 6 returned a taxable amount of income. The amounts varied from $120 to $4400. The reader must bear in mind that none of these figures represent the *gross* amount of professional income, but only the taxable part, after all legal deductions have been made.

It will be seen that the figures we have given very fully disprove the assertion frequently made, that the community pay homœopaths and quacks much better than the regular profession. A glance at the amount of taxable income returned by many of the most prominent practising lawyers in the city, also satisfied us that, as a whole, the medical profession is quite as profitable as the legal.

---

Transactions of the Illinois State Medical Society.—The volume of Transactions of our State Medical Society for 1864 is now complete and ready for distribution. Copies will be mailed to all members who have paid their dues for 1864.

The present volume is one of more than usual interest. Besides the ordinary record of proceedings at the last meeting, it contains a Report on the Epidemic Diseases and Improvements in Practical Medicine, for the year ending March 1st, 1864; by N. S. Davis, M.D., of Chicago. A Paper on Cerebro-Spinal Meningitis, by R. E. McVey, M.D., of Waverly, Ill. A Report on Orthopedic Surgery, by David Prince, M.D., of

Jacksonville. A Report on Puerperal Fever, by DeLaskie Miller, M.D., of Chicago. A Report on Surgery, by E. Andrews, M.D., of Chicago. And a Paper on "Spotted Fever," by J. A. Allen, M.D., of Chicago.

---

Illinois State Medical Society.—The next regular Annual Meeting of the Illinois State Medical Society, will be held in the City of Bloomington, on the first Tuesday in May, 1865. The Profession of the State are earnestly requested to make the meeting a full, interesting, and profitable one.

N. S. DAVIS, *Permanent Secretary.*

Chicago, Jan. 15th, 1865.

---

The Yellow Fever in Newbern, N. C.—A writer from Newbern, speaking of the great conflagration that ocurred in that city on the 19th of Nov., says: "The cause of so much property being destroyed was the complete disorganization of the once efficient fire department, the yellow fever having carried off nearly all the men, so that the engines were out of order, and the pumps nearly dry.

The yellow fever has swept away the largest portion of our population, and it is impossible to describe the scenes through which we have passed. During the rage of the pestilence all business but that of coffin-making was entirely suspended. On one single square there were thirty victims, among them Messrs. Taylor & Jones, Druggists; Robert Dunn, Moses Baer, Herritage, Oxley & Williams. In another case, out of a mess of twenty-three, only three are now living. Twelve men were appointed to bury the dead, and of these all are dead but four. Among the deaths were Wm. Moore, Colonel Avery and wife, James Bryan and wife, Mrs. Ledan, Mrs. Howard, Mrs. Allen, Mrs. Brookfield, Mrs. Osgood, Henry Jones, and his father, and Alexander Curtis.

Out of the actual residents of Newbern one hundred and twenty-five are dead. The entire number of deaths by the fever was three thousand, of course, by far the largest number being Northerners. Many of the victims were buried at night in rough coffins, and a number of the victims were found dead in their houses. The fever, as an epidemic, is now over, although some few cases occur of persons who doubtless had it in their systems. What, with fever and fire, the once beautiful town of Newbern is but the shadow of her former self.—*Medical and Surgical Reporter.*

Poisoning by Tobacco Leaves.—By Dr. Namias.—Some time ago, M. Decaisne laid before the Academy of Sciences a memoir on "The Intermittence of the Heart and Pulse occasioned by excessive Tobacco-Smoking," in which he arrived at the conclusion that the abuse can produce in certain persons a condition which may be called narcotism of the heart, and which manifests itself by intermittence in the beating of the heart and in the pulsations of the radical artery. The importance of bringing forward facts in connection with this theory induces me to record the following case:—A smuggler some months ago covered the whole of his naked body with tobacco-leaves, with the view to defrauding the revenue of the amount of the duty. The tobacco, moistend by perspiration, produced through the skin a real poisoning, which, however, was cured by means of alcoholic stimulants and laudanum. The extreme feebleness of the pulse, its smallness, the cold sweats, the fainting occasioned by the tobacco applied to the whole surface of the body, present numerous analogies with the condition called by Decaisne narcotism of the heart, and which he noticed to disappear entirely or to diminish when the use of tobacco was suspended or diminished. So far as I know, no other case of poisoning by tobacco applied to the skin has been recorded. The treatment successfully employed does not, however, lead to any general conclusions. In ordinary poisoning, the first thing is to eliminate or neutralize the poison. We must then direct attention to the condition produced by it, and this condition depends not only on the nature and quantity of the poison, but on the previous condition of the individual. We cannot thus treat poisoning by the same poison in different individuals in the same manner, because the same morbific causes do not always produce the same consecutive malady. Electric currents, which in other conditions excite hyperæmia and inflammation, only exhaust and use up directly the vital forces when they act with too great violence. I have made a similar observation as to the effect of alcoholic stimulants, which must be combated according to the different symptoms presented by the patients, that is to say, according to the different diseases which are the consequence of their abuse.—*Edinburgh Medical Journal.*

---

The Arteria Innominata Successfully Tied.—Dr. A. W. Stewart, of New Orleans, assisted by the eminent Dr. David L. Rogers, has succeeded in performing one of the most astounding feats in surgery, namely the successful ligation of the arteria innominata. The operation was undertaken in July, and the patient is now well and a monument to the fame of Dr. Mott's prediction, that it could and would be done. Besides tying this artery, the surgeons also ligated the right carotid and the right vertebral arteries to prevent the copious flow of blood arising from its return and pushing power. At one time the wound began to bleed rather profusely, but on plugging it up with *shot*, that evil was overcome.—*Phil. Med. and Surg. Reporter*.

---

Sudden Death in Contusions and Fractures from Pulmonary Emboli.—The *Med. Times and Gazette* contains an abstract from a paper, by M. Azan, before the Academy of Medicine, wherein the following conclusions were reached:—1. Fractures and contusions may give rise to sudden death through pulmonary embolism. 2. The emboli originate in a thrombosis of the veins of the injured region, itself due to the absorption of effused blood. 3. These thrombosis, or the phlebitis which precede them, are generally latent, and are of more common occurrence than would at first sight be supposed. 4. The explorations by means of the finger of the track of the veins can alone demonstrate their existence. 5. Sudden pulmonary accidents, as dyspnœa, hæmoptysis, precordial pain, syncope, etc., indications of the presence of an embolic coagulum of varying size, may direct the attention of the surgeon to the phlebitis. 6. In venous thrombosis the coagula are more or less adherent, and the plasticity of the blood is proportionate to the solidity of the adhesions. Fractures compelling repose are unfavorable to plasticity. 7. The various movements of the parts, and the application of apparatus, may favor the detachment of the coagula. 8. The surgeon should investigate whether, from the fifteenth day, in cases of fracture and contusion, latent phlebitis does not exist. 9. If this is discovered, rest, antiphlogistics, and an alkaline treatment are indicated.—*Phil. Med. and Surg. Reporter*.

---

Fibrinous Coagula of the Heart.—At a recent meeting of the London Pathological Society, Dr. Ogle presented a number of specimens illustrating the formation of fibrinous coagula in the cavities of the heart, at a long period before death. Most of these had undergone considerable softening, the centre of some of them consisting of a puriform fluid, bounded by a firm, smoothish surface like the wall of an abscess. Their firmness, color, adherence to the walls of the heart, and changes taking place with them seemed to afford conclusive evidence of their formation having occurred some time before death. As the increased frequency of the heart's action in inflammatory affections prevents the formation of these coagula, it has been suggested that a too liberal use of arterial sedatives may have the effect to favor their formation.—*Phil. Med. and Surg. Rep.*

---

Surgeon-General of New York.—We observe with much satisfaction that Dr. S. D. Willard, of Albany, has been appointed Surgeon-General of New York on the staff of the newly-elected Governor Fenton. His appointment will be well received by the profession of that State, with whom Dr. Willard is deservedly popular for his unremitting labors to promote the interest and usefulness of their noble State organization—the Medical Society of the State of New York.—*Medical and Surg. Reporter.*

---

Ligating the Arteria Innominata.—In the Reporter of September 25th, in a communication relating to the successful ligation of the *Arteria Innominata* in New Orleans it was stated that the operation was performed by Dr. A. W. Stewart. This is an error, Dr. David L. Rogers, who was present and aided in the operation, writes us: "the honor of the first successful operation on the *Arteria Innominata* belongs to Dr. A. W. Smyth, Surgeon of the Charity Hospital, New Orleans."

We make the correction with great pleasure.—*Medical and Surgical Reporter.*

---

Oxygen Gas.—At a recent meeting of the Academy of Sciences, at Munich, Baron Liebig recounted various experiments which proved clearly that oxygen is not only evolved from the atmosphere by plants, but also in tolerably large quantities by decomposition of water in the bodies of flesh-eating animals. He thinks that a knowledge of this fact will throw quite a new light on the processes of nutrition and digestion.

---

Proportion of Births to Population.—The proportion of births to population in various European countries, is given in a blue-book of "Statistical Tables relating to Foreign Countries." In England and Wales the annual births are 1 in 28 persons; 1 in 30 in Belgium, Holland, and Norway; 1 in 32 in Sweden; 1 in 33 in Hanover, the Hans Towns, and Denmark; 1 in 34 in Greece; 1 in 38 in France; 1 in 26 in Wurtemburg; 1 in 25 in Russia; 1 in 24 in Austria, Saxony, and Prussia; and 1 in 23 in Poland.

